# Lessons from Cost-Effectiveness Analysis of Smoking Cessation Programs for Cancer Patients

**DOI:** 10.3390/curroncol29100549

**Published:** 2022-09-26

**Authors:** Jeffrey S. Hoch, Heather K. Barr, Andrea M. Guggenbickler, Carolyn S. Dewa

**Affiliations:** 1 Division of Health Policy and Management, Department of Public Health Sciences, University of California, Davis, CA 95616, USA; 2 Center for Healthcare Policy and Research, University of California, Sacramento, CA 95820, USA; 3 Graduate Group in Public Health Sciences, Department of Public Health Sciences, University of California, Davis, CA 95616, USA; 4 Department of Psychiatry and Behavioral Sciences, University of California, Davis, CA 95616, USA

**Keywords:** cost-effectiveness analysis, economic evaluation, value of smoking cessation programs

## Abstract

Background: Smoking among patients diagnosed with cancer poses important health and financial challenges including reduced effectiveness of expensive cancer therapies. This study explores the value of smoking cessation programs (SCPs) for patients already diagnosed with cancer. It also identifies conditions under which SPCs may be wise investments. Methods: Using a simplified decision analytic model combined with insights from a literature review, we explored the cost-effectiveness of SCPs. Results: The findings provide insights about the potential impact of cessation probabilities among cancer patients in SCPs and the potential impact of SCPs on cancer patients’ lives. Conclusion: The evidence suggests that there is good reason to believe that SCPs are an economically attractive way to improve outcomes for cancer patients when SCPs are offered in conjunction with standard cancer care.

## 1. Introduction

It is important to understand not only the clinical consequences of adverse health behaviors (such as smoking), but also the financial and organizational consequences as well. In order to provide the best care for patients, systems operating with constrained resources (e.g., limited capacity, fixed budgets, etc.) must adapt and change. Potentially, this means embracing novel interventions to optimize outcomes given constrained resources. Cancer treatment for patients who are current smokers provides an important example of when investing in smoking cessation programs (SCPs) might be one of the most cost-effective oncology investments a healthcare system can make. Smoking among patients diagnosed with cancer poses unique health and economic burdens. Not only does active tobacco smoking (and passive exposure) account for a high amount of cancer diagnoses and deaths [1,2], uninterrupted cigarette use and smoking in diagnosed cancer patients is shown to be detrimental to both quality and length of life [3,4]. Those who continue to smoke post-cancer diagnosis often experience myriad symptoms including fatigue, coughing, symptomatic distress, and dyspnea, as well as lower self-reports of both physical functioning and mental health [3]. These symptoms pose avoidable challenges for both clinicians and patients as disease and treatment progress. Continued smoking among cancer patients has shown to be a predictor of poor prognosis, while abstinence and recent cessation have shown to be associated with significantly better prognoses [4,5]. Despite the known benefits of quitting, about a third of patients diagnosed with cancer continue to smoke [6], increasing clinical and economic burden on patients, physicians, and healthcare payers [7,8,9]. Thus, it is important to explore possible cessation options that are realistically and economically viable for patients, providers, and healthcare systems. 

Smoking cessation after a cancer diagnosis is possible and may represent a sound investment of scarce resources [10,11,12]. Time, money/funding, nursing staff, clinicians, and clinics represent just a few of the limited resources necessary in order to help curb patient, provider, and healthcare system burdens [13]. Several studies analyzing the cost-effectiveness of smoking cessation interventions in cancer patients have found that implementation of such programs is, or can be, cost-effective [14,15,16,17,18,19,20,21,22], by lowering smoking rates in cancer patients after a cancer diagnosis. Given poor prognoses in continuing smokers with cancer [4,5], smoking cessation programs stand to possibly provide cost-effective ways for healthcare entities to help patients gain quality adjusted life years (QALYs) through sustainable preventative measures. Economic evaluation allows for both mindful program assessment and usable information for advocates and decision-makers. 

This paper shares lessons about the cost-effectiveness of SCPs for cancer patients by exploring insights from a generic economic evaluation model and by reviewing findings from the scientific literature that were used to inform model assumptions. By integrating data found in the literature, our paper using decision modeling, calculates the expected value of different options, clarifying which option provides the most value. The results of these analyses can inform choices and their consequences regarding smoking cessation for future cancer patients. 

## 2. Materials and Methods

### 2.1. Methods for Cost-Effectiveness Analysis

To illustrate the types of insights that can be gained from a cost-effectiveness model, we created a simplified analysis of a smoking cessation program (SCP) for cancer patients. Any cost-effectiveness analysis (CEA) of a SCP must specify a(n): (a) cost perspective, (b) outcome choice, and (c) time horizon. If the economic evaluation results are for the purpose of informing a decision, then the “correct” costs, outcomes and time horizon should be determined in consultation with the decision-maker. For example, a cancer center would care about the costs it incurs in treatment, support, and education of patients; the Ministry of Health (MOH) might be interested in a population health outcome (e.g., quality-adjusted life years) and other healthcare costs incurred outside of the cancer center. Patients may be interested in the durability of the intervention’s effect, and patient caregivers may be interested in the resources required to support the patient at home and in the community. Our analysis assumes an MOH perspective to inform a decision of whether to invest in SCPs for cancer patients. As such, the cost perspective is that of the MOH. 

Typically, economic analyses conducted to inform MOH decisions use life years (LYs) or quality-adjusted life years (QALYs) as their outcome. In our “general” model we do not define outcome specifically but instead describe whether patients are doing “better”. The outcome “better” is used as a placeholder in this model to represent the researcher’s, or funder’s, choice of outcome (LYs, QALYs, tumor size, remission, etc.). In particular, we treat better as a binary outcome variable indicating the likelihood that patients do better after quitting. 

The study time horizon is the length of time over which costs and outcomes are considered. Ideally, a study’s time horizon is chosen based on the expected length of time over which a new intervention affects cost and outcome. Frequently when studying the value of new cancer treatments or interventions, it is not possible to collect data for the number of years over which the new intervention continues to affect cost and outcome. While our simple model does not specify a time-horizon, it is important to note that exact estimates of cost-effectiveness can vary with choice of time horizon. More complicated models with longer timeframes often allow a new intervention to demonstrate greater effectiveness (and potentially greater cost). 

As a whole, the expected effectiveness of SCPs relies on two key parts: (1) reduction in smoking; (2) benefits from reduction in smoking. Table 1 summarizes the notation for our analysis. The probability that a cancer patient quits smoking with a SCP is 𝒴, and the probability that a cancer patient quits smoking without a SCP is Y. For simplicity, we assume that the probability that a cancer patient has a better outcome with treatment if they quit smoking is y, which does not depend on why the patient quit (e.g., because of the SCP or not). The probability that a cancer patient does not quit smoking with a SCP is 𝓝 = 1 − 𝒴. Likewise, the probability that a cancer patient quits smoking without a SCP is N = 1 − Y. 

**Figure 1:**
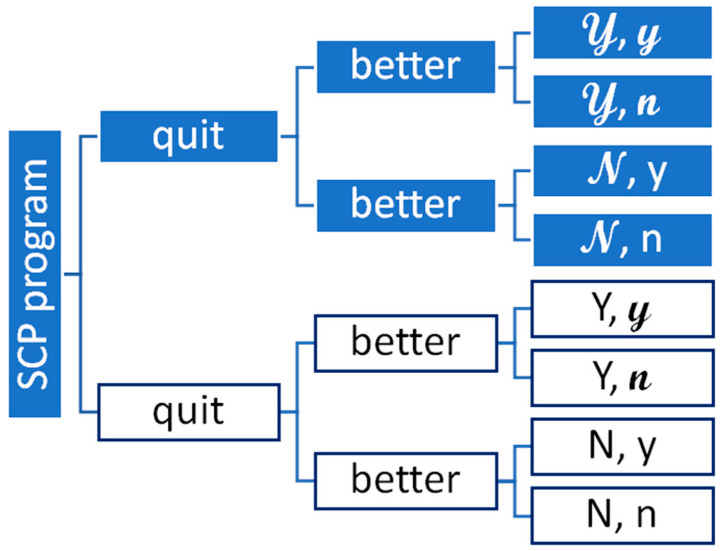
Diagram of the key parts in an economic evaluation of a smoking cessation program. Note: Figure 1 illustrates a “general” decision tree used to calculate the expected value of a smoking cessation program (SCP) vs. no SCP with two “chance” events: (1) people quit; and (2) quitting leads to better outcomes. The SCP option has shaded boxes and the no SCP option does not.

Figure 1 illustrates the key parts of our cost-effectiveness model. Figure has shaded (for the SCP) and unshaded (for not having the SCP) boxes. The rightmost part of Figure 1 features eight boxes denoting all potential outcomes, using the notation in Table 1. For example, the top box on the right of Figure 1, marked as 𝒴,𝔂, represents the likelihood of a patient quitting and having a better outcome with a SCP. In contrast, the bottom box, marked as N,n, represents the chance of a patient not quitting smoking and not having a better outcome.

### 2.2. Methods for Review of the Scientific Literature

The model uses data inputs, and we sought estimates from the published peer-reviewed literature. The PubMed database was searched for cost-effectiveness analyses of SCPs with the search terms: smoking cessation, cancer diagnosis, cost effectiveness, cancer prognosis, and cost-effective analysis. The articles were limited to the English language with models focusing on the United States or Canada. This search yielded 122 articles. To be included for this paper, the article needed to focus on cost-effectiveness of SCP run in-tandem with cancer treatment (3 articles) [17,18,21] or at the time of cancer screening (4 articles) [15,16,19,22]. An additional search of Google Scholar using the same terms was independently conducted and screened by an additional team member. No additional published articles were found. 

Data extraction from the papers was a multistep process. When given, the incremental cost effectiveness ratio (ICER) for QALYs was extracted. If the ICER for a QALY was not available in the paper, our team calculated the values using information on program costs and effects that were provided. Costs were converted into 2022 monetary units and then to Canadian dollars using a country-specific consumer price index and currency conversion process where necessary. Data for the extra costs and extra effects (i.e., QALYs), of SCPs were extracted to examine constituent components of the ICER (e.g., is the ICER large because of small QALY gains or large additional costs). We separated our CEA studies of SCPs based on two settings: (1) SCPs for cancer patients; and (2) SCPs added to cancer screening programs.

## 3. Results

In this section, we present the two parts of our results. We begin with the results from the model. This is followed by the results of our review. 

### 3.1. Results from the Cost-Effectiveness Model

As noted in Section 2.1, calculating the expected value of an economic model involves comparing the smoking cessation program (SCP) “payoff” (i.e., 𝒴,𝔂 + 𝒴,𝓃 + 𝒩,y + 𝒩,n) to the no SCP “payoff” (i.e., Y,𝔂 + Y,𝓃 + N,y + N,n). By treating, the outcome of “better” as a binary variable, the values to be compared are 𝒴,𝔂 + 𝒩,y versus Y,𝔂 + N,y. As noted earlier, 𝒩 = 1 − 𝒴 and N = 1 − Y. Therefore, the expected value of a SCP is calculated as 𝒴 × 𝔂 + (1 − 𝒴) × y and the expected value of not having a SCP corresponds to Y × 𝔂 + (1 − Y) × y. The difference between them is

𝒴 × 𝔂 + (1 − 𝒴) × y − [Y × 𝔂 + (1 − Y) × y] = 𝒴 (𝔂 − y) – Y (𝔂 − y) = (𝒴 − Y) (𝔂 − y).

The first term (𝒴 − Y) is the additional effectiveness of a SCP in getting patients to quit and the second term (𝔂 − y) is the additional effectiveness of quitting, helping patients have better outcomes. Thus, the expected value of a SCP compared to not having one equals the product ΔE_1_ ΔE_2_ where we define ΔE_1_ = (𝒴 − Y) and ΔE_2_ = (𝔂 − y). 

**Figure 2:**
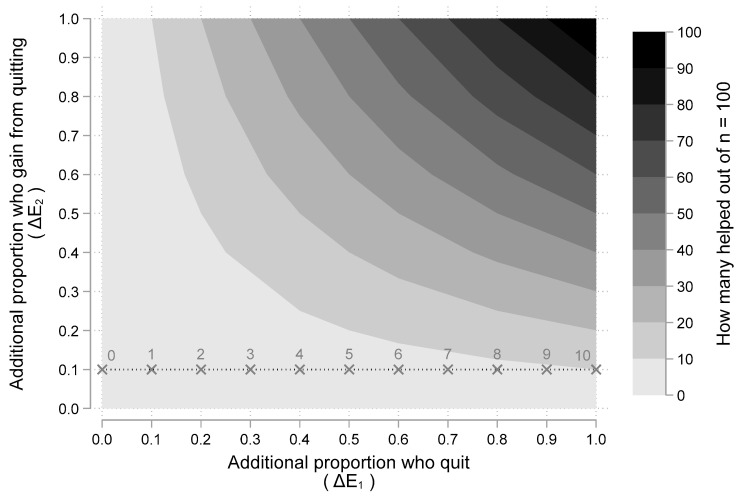
How many people from a population of n = 100 are helped by a smoking cessation program as a function or the proportion who quit and the proportion who benefit from quitting. Note: Figure 2 illustrates a “two-way” sensitivity analysis illustrating the impact of different assumptions about the two main “chance” events: (1) additional proportion of people who quit (i.e., ΔE_1_); and (2) additional proportion who gain better outcomes from quitting (i.e., ΔE_2_). The shaded area indicates how many people will benefit out of a hypothetical population of 100 given assumed values for ΔE1 and ΔE_2_. At ΔE_2_ = 10%, 10% increases in ΔE_1_ increases the number of people helped by one (as indicated by the labels over the points marked with an ×. At ΔE_2_ = 100%, 10% increases in ΔE1 increases the number of people helped by ten (as indicated by the shaded regions).

We illustrate the relationship derived above by considering a cancer patient population of 100 people (n = 100) who smoke. How many would be helped with a SCP? Figure 2 illustrates the answer using the expected value equation ΔE_1_ ΔE_2_ derived above. The ten shaded regions indicate how many people are helped. The lightest shaded region, from 0–10 people helped, appears in the lower left of Figure 2, while the darkest shaded region from 90–100 people helped, appears in the upper right of the figure. As either the proportion who quit (ΔE_1_) or the proportion who benefit from quitting (ΔE_2_) increase, the amount of people helped increases. An important insight is that the impact of increasing ΔE_1_, depends on the assumed value for ΔE_2_.

For example, if we assume that everyone who quits smoking has a better outcome (i.e., ΔE_2_ = 1.00), then increasing the proportion who quit by 10%, increases the number of people benefited by 10. However, if we assume that only 10% who quit smoking have a better outcome (i.e., ΔE_2_ = 0.1), then increasing the proportion who quit by 10%, increases the number of people helped by 1. This scenario is illustrated in Figure 2 with the dashed horizontal line at ΔE_2_ = 0.1. For various values of ΔE_1_, the number of people helped is labeled and marked with an “X”. The dashed line illustrates that for every 10% increase in the proportion who quit, one more person is helped (as the numeric labels above each “X” increase by 1). On the other hand, if the horizontal line were at ΔE_2_ = 1, for every 10% increase in the proportion who quit, ten more people would be helped (as indicated by the shaded regions). Thus, the impact of additional quitting (ΔE_1_) depends on the additional benefits patients gain from quitting (ΔE_2_). 

**Figure 3:**
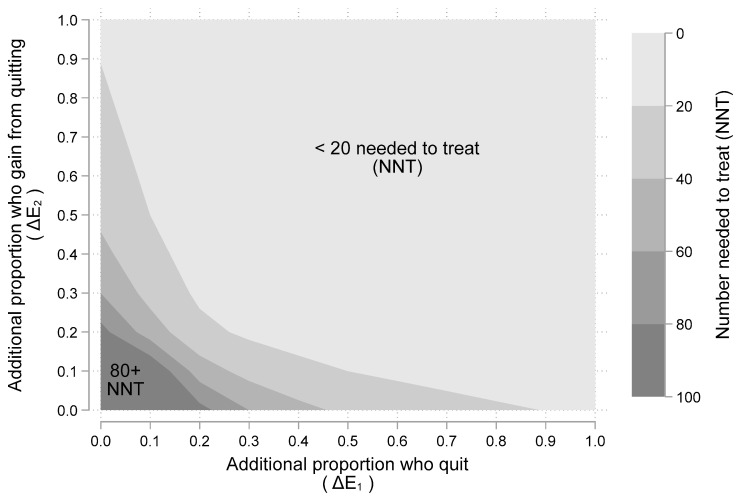
The “number needed to treat” (NNT) with a smoking cessation program for one more person to benefit. Note: Figure 3 illustrates a “two-way” sensitivity analysis illustrating the impact of different assumptions about the two main “chance” events: (1) additional proportion of people who quit (i.e., ΔE_1_); and (2) additional proportion who gain better outcomes from quitting (i.e., ΔE_2_). The shaded areas indicate number needed to treat (NNT) given assumed values for ΔE1 and ΔE_2_. Larger NNT values require more smokers being “treated” with a smoking cessation program (SCP) before one is helped.

Figure 3 show another view of the gains from a SCP as a function of additional quitting (ΔE_1_) and additional benefits gained from quitting (ΔE_2_). The five shaded regions indicate the number needed to treat (NNT) [23,24], with SCPs for one person to benefit (i.e., have a better outcome). The lightest shaded region, from 0–20 people needed, appears in the upper right of Figure 3, while the darkest shaded region from 80–100 people needed, appears in the lower left of the figure. As an example, if a SCP leads to 20% more quitting (i.e., ΔE_1_ = 0.2) and quitting leads to 50% chance of better outcomes (i.e., ΔE_2_ = 0.5), then the number needed to treat is ten (i.e., NNT = 1/(0.2 × 0.5) = 1/0.10 = 10). From Figure 3, it appears that even very modest assumptions about smoking cessation from SCPs and their subsequent benefits can lead to reasonably small NNTs. Results from the scientific literature presented next offer reasonable values to assume for ΔE_1_ and ΔE_2_. 

### 3.2. Results from the Review of Scientific Literature

**Figure 4:**
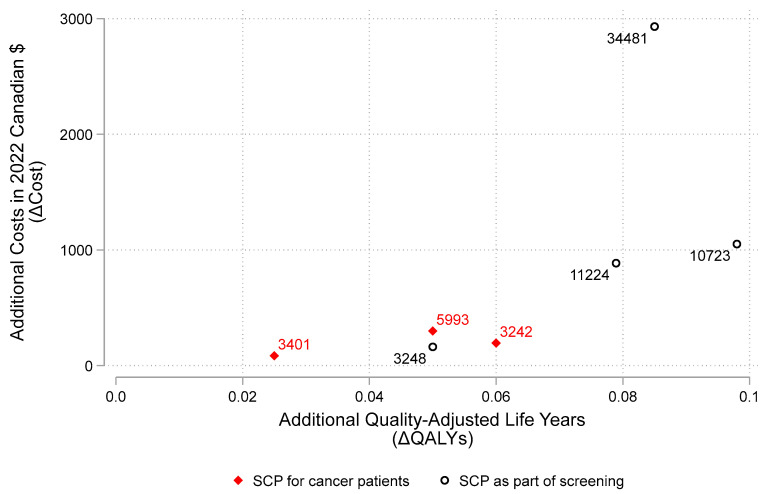
Cost-effectiveness plane. Note: Figure 4 illustrates results reported in the peer-reviewed scientific literature on smoking cessation programs (SCPs) estimating the extra quality-adjusted life years (ΔQALYs) and the extra costs in 2022 Canadian dollars (ΔC). The ratio ΔC/ΔE (i.e., the incremental cost-effectiveness ratio or ICER) is shown as the label for each point. Diamond points (i.e., ♦) show results from studies of SCPs for cancer patients while circular points (i.e., ○) show study results for SCPs supplementing cancer screening programs.

The model recognizes the impact of improved cessation with SCPs in the form of patients doing “better” (e.g., this can be envisioned as improvement in life years (LYs) or quality adjusted life years (QALYs)). Our literature review provides costs and outcomes information based on research about SCPs among cancer patients. Figure 4 plots the additional effectiveness (in QALYs) against the addition costs (in 2022 Canadian dollars) based on our review the scientific literature [15,16,17,19,21,22]. Diamond points (i.e., ♦) show results from studies of SCPs for cancer patients while circular points (i.e., ○) show study results for SCPs supplementing cancer screening programs. While all of the points are to the right of zero (indicating having a SCP is more effective than not), the SCPs as part of a screening program appear to have the potential for greater QALY gains. This is plausible given a SCP as part of a screening program would have more time over which to effect change. The ratio ΔC/ΔE (i.e., the incremental cost-effectiveness ratio or ICER) is shown as the label for each point in Figure 4. Most studies show SCPs for people with cancer have extra costs around $1000 or less with ICER estimates averaging around $6900 (median = $3458); ICERs for SCPs as part of a cancer screening program average around $20,484 (median = $13,732). 

**Figure 5:**
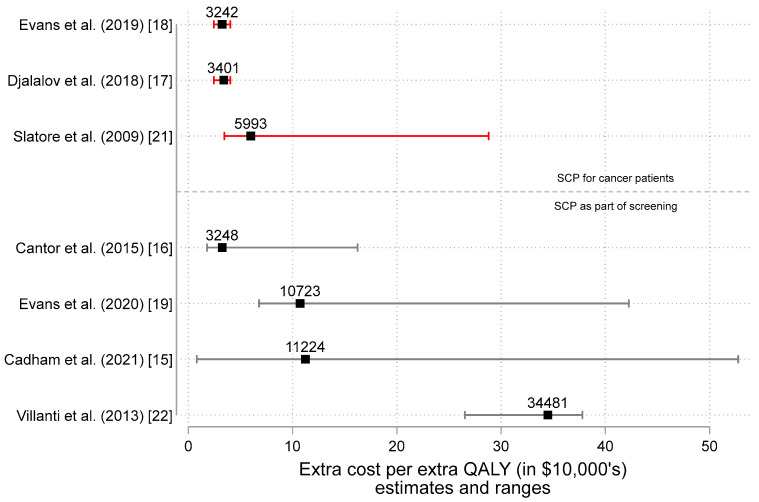
Estimates of the incremental cost-effectiveness ratio (ICER = ΔC and ΔE estimates) in 2022 Canadian dollars by study type [15,16,17,18,19,21,22]. Note: Figure 5 illustrates the estimates and ranges for the extra cost per extra QALY (in $10,000′s) as reported in the peer-reviewed scientific literature on smoking cessation programs (SCPs). The ratio ΔC/ΔE (i.e., the incremental cost-effectiveness ratio or ICER) is shown as the label for each point. Results from studies of SCPs for cancer patients appear above while study results for SCPs supplementing cancer screening programs appear below. Missing from the studies of SCPs as part of screening are the results from Goffin et al. (2016) [20]. Interested readers are directed to Table 2 in the Goffin et al paper for 14 different estimates conveying that SCPs are more effective and more costly).

Figure 5 illustrates the range of the ICER estimates plotted in Figure 4. For example, after adjusting for inflation and converting currency to CAN$, Cadham et al. (2021) estimates the range of cost per QALY to be as low as $823.77 to upwards $52,737.85, depending on how intensive the program is [15]. Overall, we estimate the ICER for a SCP among cancer patients could range from 3–30,000, though could reach as high as $50,000 when added to a cancer screening program. 

## 4. Discussion

### 4.1. Integrating Methods with Published Work: Effectiveness, Cost and Cost-Effectiveness

#### 4.1.1. Effectiveness

The results from our general modeling exercise demonstrated that gaining “nothing” from SCPs occurs if either ΔE_1_ or ΔE_2_ is zero. These scenarios happen when the couplet (ΔE_1_, ΔE_2_) is located on the vertical or horizontal axis of Figure 2 (or Figure 3). For example, if 25% of people in a smoking cessation program (SCP) quit but there is no benefit from quitting (i.e., ΔE_1_ = 0.25 and ΔE_2_ = 0.0), then there is no expected gain (since ΔE_1_ × ΔE_2_ = 0). Another example occurs in Figure 2, where there is an “X” at the point (0, 0.1). This corresponds to a SCP having zero chance of improving quit rates paired a 10% chance that patients who quit will have better outcomes. This point in Figure 2 is labeled 0 since there is no expected gain from a SCP. However, once one assumes that there is at least some gain both in terms of (1) less smoking and (2) some benefit from less smoking, the expected value is positive and having a SCP is more effective than not. 

Based on our literature search we found evidence suggesting that it is likely neither ΔE_1_ nor ΔE_2_ is zero. For example, for ΔE_1_, there is recent evidence from a cost-effectiveness of implementing smoking cessation interventions for patients with cancer [14]. Levy et al. (2022) used data from the Smokefree Support Study (conducted 2013–2018; completed 2021), to estimate quit rates between 21.4% and 34.5% with a SCP. The quit rate of 14.3% for “usual care” came from Park et al. [25]. These estimates are congruent with a low ΔE_1_ estimate of 7.1% (i.e., 21.4–14.3) or high estimate of 20.2% (i.e., 34.5–14.3). For SCP enthusiasts believing that the program will have a 100% quit rate, the theoretically highest ΔE_1_ estimate is 85.7% (100–14.3). For usual care pessimists who believe no cancer patient would quit smoking without a SCP, a reasonable range for the ΔE_1_ estimate would be 21.4% (21.4–0) to 34.5% (34.5–0). Therefore, the scientific evidence supports the belief that the estimate ΔE_1_ > 0. Having argued that ΔE_1_ is likely bigger than zero, we next focus on whether it is reasonable to assume ΔE_2_ > 0. This is important because if both ΔE_1_ and ΔE_2_ > 0, then having a SCP is more effective than not.

The literature consistently supports the notion that ΔE_2_ > 0. For example, Evans et al. (2019) notes that, “The evidence that smoking cessation improves cancer treatment outcomes is irrefutable.” Referencing the 2014 US Surgeon General’s report [26] summarizing the evidence, Evans et al. (2019) [18] reminds readers that the report concluded that, “continued smoking after a diagnosis of cancer can result in an increase in all-cause and cancer-specific mortality, greater toxicity from therapeutic interventions and an increased incidence of recurrence and second malignancies.” In addition, all of the Δquality adjusted life year (QALY) estimates in Figure 4 appear to the right of 0; this is evidence that SCPs are improving either quality, length of life or both. This cannot occur unless ΔE_2_ > 0. The amount of ΔQALYs gained from SCP depends on context and data inputs selected from the scientific literature; however, there appears to be a consistent message in favor of the effectiveness of SCPs. 

#### 4.1.2. Cost and Cost-Effectiveness

Nevertheless, one cannot conclude SCPs fare cost-effective until examining the additional costs as well. While there are many different types of SCPs, most impose little extra cost (i.e., ΔC is small). For example, while most studies show SCPs have extra costs around $1000 or less, the ratios of extra cost to extra effect (i.e., the incremental cost effectiveness ratios or ICERs) appear quite reasonable. ICERs for SCPs for cancer patients average around $4200 (median = $3400) and ICERs for SCPs as part of a cancer screening program average around $15,000 (median = $11,000). These are incredibly “good investments” (i.e., gains in patient outcomes for relatively low extra cost) compared to almost anything in the oncology portfolio, especially new treatments [27]. Assuming a gain of 0.02 QALYs (lower than most of what has been published) from SCPs for cancer patients, a new SCP would need to cost around $20,000 per person to reach a $100,000 per QALY threshold commonly found in oncology. Given that both the extra health gain is more than 0.02 QALYs and the extra costs are likely less than $20,000 per person, SCPs are likely cost-effective. 

### 4.2. Other Issues

However, there are other factors to be considered as well when funding SCPs. A major limitation in our analysis is that we have omitted consideration of key issues outside of the immediate extra effectiveness (ΔE) and the extra cost (ΔC) of SCPs. Issues related to implementation and continued funding deserve more attention. It is our belief that these concerns do not preclude funding of SCPs; however, attention to them will greatly enhance the value of any investment in SCPs. There are other benefits of SCPs that were not included in our model. For example, smoking cessation may make other cancer treatments more cost-effective by making them more effective. Furthermore, smoking cessation may reduce other healthcare problems as well as may lead to fewer cancers due to secondhand smoke. 

## 5. Conclusions

For both theoretical and applied perspectives, there is strong reason to believe smoking cessation programs (SCPs) for cancer patients are cost-effective. In fact, given the incremental cost-effectiveness ratios (ICERs) reported in the scientific literature, their value may be greater than most new cancer treatments. It is easy to believe the value of SCPs remains when they are associated with cancer screening programs, based on peer-reviewed publications. More expensive, less effective investments are common in oncology. It seems reasonable that healthcare payers might invest in supporting SCPs and studying their value as part of real-world evidence building process. They could use this process to address any lingering doubts that are presently inhibiting wider uptake of this incredibly cost-effective way to help people with cancer and their loved ones.

## Figures and Tables

**Table 1:** Notation for quitting probabilities as well as doing better probabilities by program *.

	Probability of	
**Option**	Quitting	Not Quitting
SCP Program	𝒴	𝓝
No SCP program	Y	N
**After**	Doing Better	Not Doing Better
Quitting	𝔂	𝓃
Not quitting	y	n

* Note: SCP = smoking cessation program.

## Data Availability

The data presented in this study are available on request from the corresponding author.
